# Shape Optimization for Additive Manufacturing of Removable Partial Dentures - A New Paradigm for Prosthetic CAD/CAM

**DOI:** 10.1371/journal.pone.0132552

**Published:** 2015-07-10

**Authors:** Junning Chen, Rohana Ahmad, Hanako Suenaga, Wei Li, Keiichi Sasaki, Michael Swain, Qing Li

**Affiliations:** 1 School of Aerospace, Mechanical and Mechatronic Engineering, the University of Sydney, Sydney, NSW 2006, Australia; 2 Unit of Prosthodontics, Faculty of Dentistry, Shah Alam & Integrative Pharmacogenomics Institute (iPROMISE), Universiti Teknologi MARA, Bandar Puncak Alam, Selangor, 42300, Malaysia; 3 Division of Preventive Dentistry, Tohoku University Graduate School of Dentistry, 4–1 Seiryo-machi, Aoba-ku, Sendai, 980–8575, Japan; 4 Division of Advanced Prosthetic Dentistry, Tohoku University Graduate School of Dentistry, 4–1 Seiryo-machi, Aoba-ku, Sendai, 980–8575, Japan; 5 Faculty of Dentistry, The University of Sydney, Sydney, NSW 2006, Australia; Department of Biomaterials, JAPAN

## Abstract

With ever-growing aging population and demand for denture treatments, pressure-induced mucosa lesion and residual ridge resorption remain main sources of clinical complications. Conventional denture design and fabrication are challenged for its labor and experience intensity, urgently necessitating an automatic procedure. This study aims to develop a fully automatic procedure enabling shape optimization and additive manufacturing of removable partial dentures (RPD), to maximize the uniformity of contact pressure distribution on the mucosa, thereby reducing associated clinical complications. A 3D heterogeneous finite element (FE) model was constructed from CT scan, and the critical tissue of mucosa was modeled as a hyperelastic material from *in vivo* clinical data. A contact shape optimization algorithm was developed based on the bi-directional evolutionary structural optimization (BESO) technique. Both initial and optimized dentures were prototyped by 3D printing technology and evaluated with *in vitro* tests. Through the optimization, the peak contact pressure was reduced by 70%, and the uniformity was improved by 63%. *In vitro* tests verified the effectiveness of this procedure, and the hydrostatic pressure induced in the mucosa is well below clinical pressure-pain thresholds (PPT), potentially lessening risk of residual ridge resorption. This proposed computational optimization and additive fabrication procedure provides a novel method for fast denture design and adjustment at low cost, with quantitative guidelines and computer aided design and manufacturing (CAD/CAM) for a specific patient. The integration of digitalized modeling, computational optimization, and free-form fabrication enables more efficient clinical adaptation. The customized optimal denture design is expected to minimize pain/discomfort and potentially reduce long-term residual ridge resorption.

## Introduction

Denture treatments have been widely applied in dental practice to restore oral function of the edentulous group [1]. In all cases of tissue-borne, tooth-tissue-borne, and implant-retained dentures, the oral mucosa plays a critical role in distributing occlusal loads from the denture to the underlying bony ridge during mastication [2–5]. The contact pressure developed over this highly vascular tissue indicates one of the most important etiological factors causing the clinical complications [5–8] and highly correlated with prognosis [9]. Excessive contact pressure under the denture disturbs local blood supply to the underlying bone, triggering nerve pain and discomfort in patients [9–11], thus compromising their quality of life [12]. In more severe cases, subsequent residual ridge bone resorption may develop [13, 14].

Contact pressure distribution can be affected by the anatomy and physiological responses of both residual mucosa and underlying jaw bone [2–4, 6, 7, 15], leading to distinctive patterns in the individual patients. Conventional denture fabrication using final cast models [16–19] is unable to take into account either the nonlinear soft tissue responses or the heterogeneous jaw bone with local variations, and the fabricated denture base reflects only surface morphology of residual ridge. Meanwhile, this labor-intensive process may result in variable adaptive accuracies with different fabrication techniques and experience levels [16].

The finite element (FE) method has demonstrated its compelling advantages for biomechanical analysis and surgical planning [20–22]. When combined with clinical computed tomography (CT), precise 3D morphologies can be modeled properly with both anatomical and physiological features of an individual patient [1]. Increasing computational power enables to more realistically modelling tissue behavior, including complex nonlinear biomaterial responses [20]. Furthermore, computational design allows optimizing the shape of an engineering structure, and has been applied for addressing design issues involving sophisticated contact conditions [23–26]. Following the success achieved in engineering, the denture base can potentially be modified and adjusted pre-fabrication and pre-clinical try-in to avoid possible excessive contact pressure over the underlying mucosa to minimize potential clinical complications [27]. This computational approach also facilitates various digitalized additive manufacturing, forming a CAD/CAM procedure for ensuring controllable consistency and standardized accuracy [28–30].

This study aims to develop a fully automatic computational algorithm for denture base design in a patient-specific removable partial denture (RPD) by increasing the uniformity of contact pressure, followed by additive prototyping and *in vitro* verification. A 3D heterogeneous FE model of the patient’s mandible with teeth and RPD is first created based on clinical CT data, with the mucosa modeled as a nonlinear (hyperelastic) material in response to mastication. Both initial and optimized dentures were prototyped by 3D printing technology, which were subsequently tested with pressure sensitive silicone and films, to verify the effectiveness of the proposed procedure *in vitro*. Both pressure-induced pain and hydrostatic pressure in the mucosa are examined with the initial and optimal contact surfaces against clinical data reported in literature. From biomedical perspective, this study develops a novel procedure by the integration of FE based automatic design optimization and additive fabrication (3D printing). From a clinical perspective, this novel technique automates patient-specific denture fitting with quantitative guidelines for denture adjustment and correction in a more efficient way. From a patient perspective, the optimized denture would potentially minimize pain/discomfort and reduce long-term residual ridge resorption, thereby maximizing intervals between further adjustments of denture.

## Materials and Methods

### Patient data acquisition and modeling

**Fig 1:**
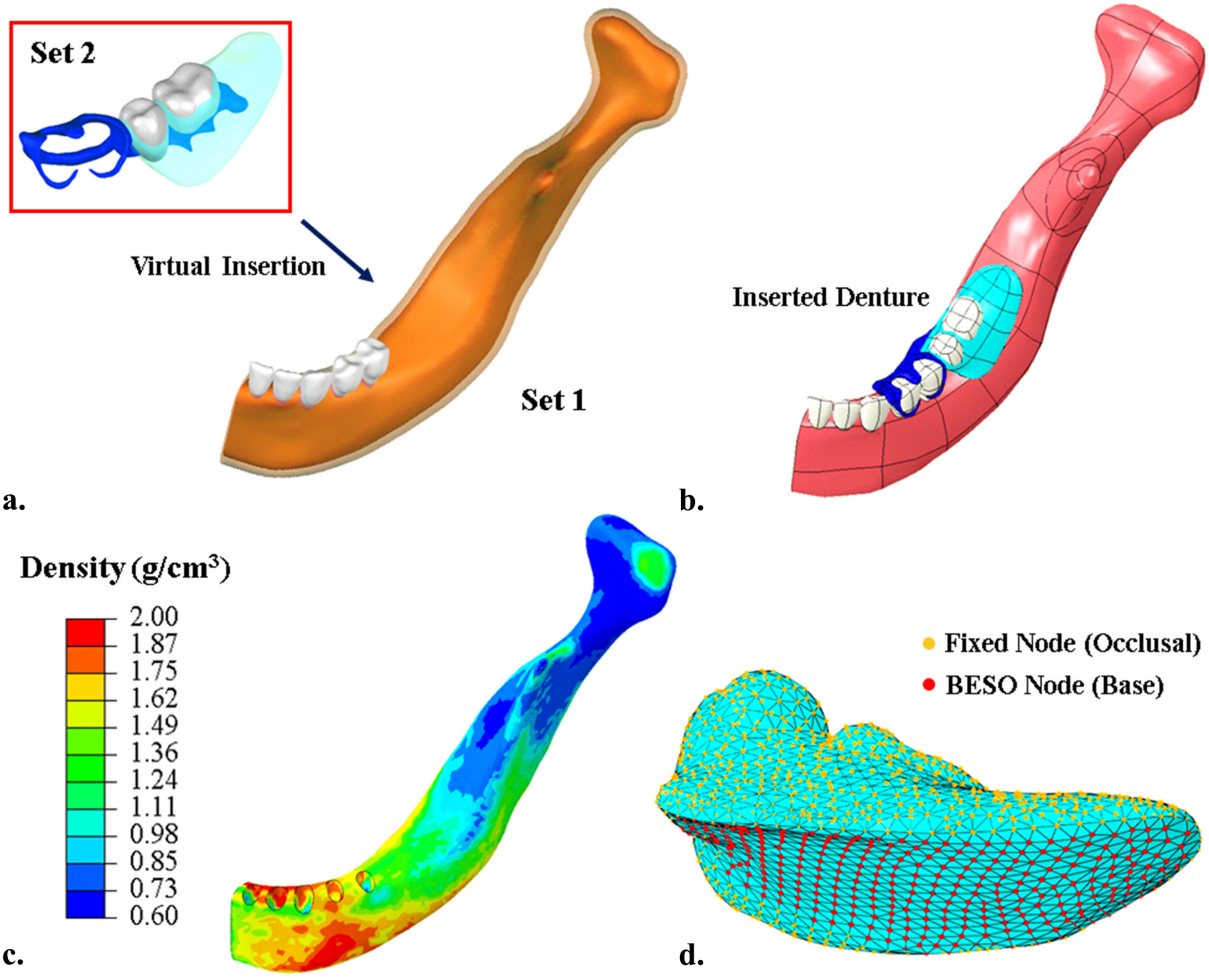
The model was created based on patient-specific clinical data, a) two sets of CBCT data were reconstructed to form masks (Set 1: white—teeth, orange—bone, transparent pink—mucosa, grey—teeth; Set 2: grey—artificial teeth, transparent cyan—denture base, blue—denture frame); b) both models are solidified by using NURBS and assembled; c) assignment of heterogeneous material property of the bone based on HU value; d) the nodes highlighted in red on the denture base are allowed to be modified during bi-directional evolutionary shape optimization (BESO), and the orange ones are fixed to maintain the denture functionality.

The research protocols and a patient consent form were approved by the research ethics committee at both the Tohoku University Graduate School of Dentistry and Sendai Kousei Hospital. For this study, a written patient consent form was provided and signed by the participant for utilizing the image data. The specific subject was a female at early 60s, and her left mandible with partially edentulous area was reconstructed up to the midsagittal plane from the clinical CT scans with a resolution of 0.3 mm per pixel, for the region of interest and computational efficiency, in ScanIP Ver. 4.3 (Simpleware Ltd, Exeter UK) [31]. The geometric models were then created by using non-uniform rational B-splines (NURBS) in the 3D parametric modeling software Rhinoceros 3.0 (Robert McNeel & Associates, Seattle USA) (Fig 1a). The experimental RPD was made with a titanium frame, a scanning resin denture base, and radiopaque artificial teeth, for the scanning purpose, and modeled for virtual insertion through the surface contour registration between the rest seats on the abutment teeth and the rest of RPD components in Rhinoceros (Fig 1b). This initial model provides a baseline for the base contact without mastication prior to optimization.

The FE model was meshed in ABAQUS 6.9.2 (Dassault Systèmes, Waltham USA), with a mesh convergence test to ensure sufficient numerical accuracy [32, 33]. The final mesh contains 186 213 degrees of freedom (D.O.F.) with 328 066 tetrahedral elements in the hybrid formulation (C3D4H) to preserve the smoothness of the contact surfaces in the nonlinear analysis.

### Material assignment and loading scenario

The common assumptions of linear elastic and homogenous materials [34, 35] may not adequately replicate complex tissue responses [20]. In this study the mandibular bone was more realistically characterized as a heterogeneous material based on the HU values [36], reflecting the variation of localized modulus and load deflection [34, 37], as well as soft tissue response on the surface [20]. The apparent bone density *ρ*_*app*_ is interpolated linearly against the HU value as in Eq (1), with the thresholds of 1.86 g/cm^3^ and 0.72 g/cm^3^ for cortical and cancellous bones [38] (the resultant bone density contour in Fig 1c). Eq (2) was adopted here to correlate the Young’s modulus *E* to the apparent density *ρ*_*app*_ [39], and a User Defined Field (USDFLD) subroutine was employed to assign heterogeneous material properties in ABAQUS, where the rate-dependent term, ${\dot{\varepsilon }}_{e}^{0.06}$, was assumed to be approaching 1 as the low speed loading in the normal chewing activities. For the simplification of the simulation, the isotropy of the mandibular bone is assumed [34, 36, 37, 40], as its directional difference is much less significant compared to its heterogeneity and the soft tissue mucosa.

$$\rho_{app}=\rho_{\text{min}}+(\rho_{\text{max}}-\rho_{\text{min}})\times \frac{\left(HU-HU_{\text{min}}\right)}{\left(HU_{\text{max}}-HU_{\text{min}}\right)}$$ (1)

$$E=3790{\dot{\varepsilon }}_{e}^{0.06}\rho_{app}^{3}$$ (2)

$$U_{\varepsilon }=\overset{M}{\underset{i=1}{\sum }}\frac{2\mu_{i}}{\alpha_{i}^{2}}({\bar{\lambda }}_{1}^{\alpha_{i}}+{\bar{\lambda }}_{2}^{\alpha_{i}}+{\bar{\lambda }}_{3}^{\alpha_{i}}-3)+\overset{N}{\underset{i=1}{\sum }}\frac{1}{D_{i}}{\left(J^{\varepsilon l}-1\right)}^{2i}$$ (3)

A hyperelastic constitutive material model was adopted for the mucosa to better represent its nonlinear response under mechanical loading [3, 41, 42]. This material model depends upon the strain energy (*U*_*ε*_) stored per unit volume as a function of the instantaneous volume strain (*J* ^*εl*^) at a point of the material, and was derived from the clinical data [42] via the least-squares fitting. The third order Ogden strain energy constitutive model equation [43] was determined to provide the closest match (Eq 3) with the least complexity, where ${\overset{-}{\lambda }}_{i}$ are the deviatoric principal stretches obtained from the principal stretches, *M* (= 3 in this case) is the order of this fitting equation. Table 1 summarizes all the material properties, including *μ*_*i*_, *α*_*i*_, and *D*_*i*_ for the hyperelastic model [1, 34, 35].

**Table 1:** Material properties of the denture and mandible.

Material	Young Modulus (MPa)		Poisson Ratio	
**Bone**	Heterogeneous		0.30	
**Denture Frame (Titanium)**	110 000		0.35	
**Denture Base (Acrylic)**	2 650		0.30	
**Artificial Tooth (Porcelain)**	140 000		0.28	
**Tooth**	84 100		0.20	
**Mucosa**	**Hyperelastic**		0.47	
***(Ogden 3^rd^)***	*i*	*μ_i_*	*α_i_*	*D_i_*
	1	3.26E-02	8.41	12.47
	2	7.88E-04	25.00	0
	3	1.03E-03	-18.94	0

In this study, the unilateral biting is considered as the critical activity in mastication. To simulate this loading scenario, a localized masticatory force was applied in the vicinity of the first molar approximately along the tooth axis with a magnitude of 130 N [40, 44]. Different masticatory activities (e.g. grinding) with various loading patterns may affect the optimization outcome, but their effects are less significant compared to the direct biting because of the magnitudes [23]. The boundary conditions were prescribed to the distal condyle with full kinematic constraints and the sagittal plane with symmetric constraints [45, 46]. A perfect clasp fitting was assumed for this simulation to reduce computational load with a focus on the mucosa-denture interface. The Augmented Lagrangian algorithm was adopted to simulate the denture-mucosa contact, with a low frictional coefficient assumed at 0.1 to mimic typical lubrication in the oral environment [47, 48, 49].

### Bi-directional evolutionary structural optimization (BESO)

The BESO algorithm is a heuristic and non-gradient approach [23] making adjustment to a structure by progressively adding material to where it is most needed, and removing material from where it is most redundant in a concurrent manner. Conventional element-based BESO [23, 50] leads to non-smooth zigzag boundaries, and hence it is modified significantly here by using a cloud of surface nodes, rather than individual elements, as the design domain. This preserves a functional shape for a realistic contact. The surface mesh in Fig 1d indicates the boundary nodes in the design domain, where two groups of such nodes are highlighted in red (i.e. free-to-move nodes and capable of being optimized) and orange (constrained to maintain the functionality of a denture).

**Fig 2:**
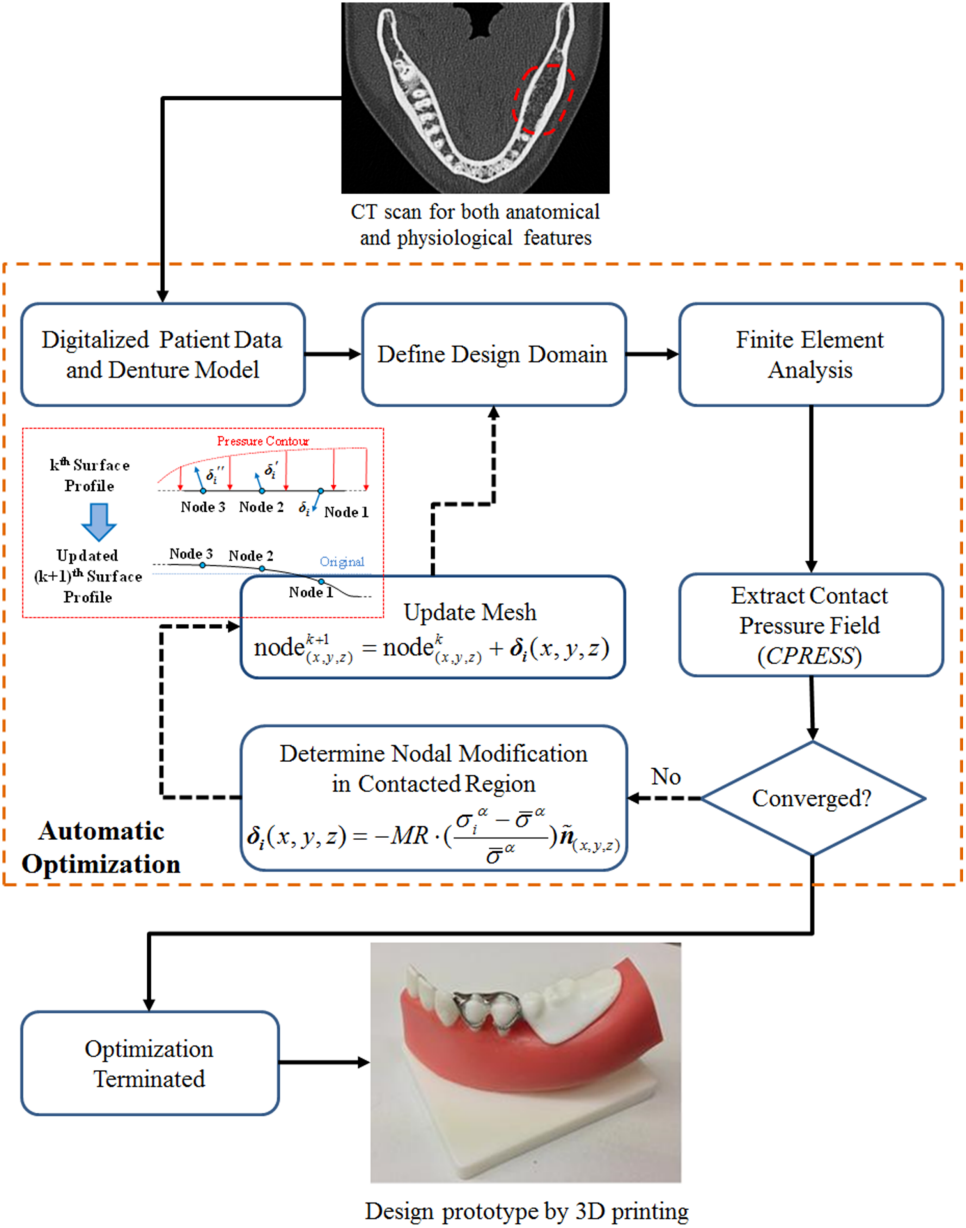
The flow chart illustrates the contact-based BESO procedure for optimization of denture contact surface.

The clinical expectation is to avoid undesirable contact stress concentrations over the entire contact profile (denoted as *g*(*x*,*y*,*z*) in Eq (4)). This can be achieved by reducing the overall deviation (*F* in Eq (4)) of the contact pressure, which reduces the magnitude of the upper extremes and improves the effectiveness of the lower extremes. *N* denotes the total number of nodes in the design domain; *σ*_*i*_ is the nodal contact pressure, and $\overset{-}{\sigma }$ is the average contact pressure in each iteration (*k =* 1,2,*…K*). The optimization criterion is considered convergent when the average change of objective function over 5 contiguous iterations is less than 1% (Eq (5)). The interfacial contact surface *g* is modified by implementing nodal movement ***δ*** as Eq (6), and an adaptive modification rate ($MR_{Adaptive}^{\left(k\right)}$) controlled by the maximum contact pressure ratio as defined in Eq (7), where the initial modification rate is set to *MR* ^(0)^ = 1% herein and the subsequent modification rate is defined by the ratio between the iterative maximum and the initial maximum contact pressures. The whole contact-based BESO procedure is depicted in the flowchart shown in Fig 2.

$$\text{min}F=\;f^{\left(k\right)}\left[g(x,y,z)\right]=\sqrt{\frac{1}{N}\sum_{i=0}^{N}{\left(\sigma_{i}-{\bar{\sigma }}^{\left(k\right)}\right)}^{2}}$$ (4)

$$\;\tau =\frac{\overset{K}{\underset{k=K-4}{\text{max}}}f^{\left(k\right)}\left[g(x,y,z)\right]-\overset{K}{\underset{k=K-4}{\text{min}}}f^{\left(k\right)}\left[g(x,y,z)\right]}{\frac{1}{5}\sum_{k=K-4}^{K}f^{\left(k\right)}\left[g(x,y,z)\right]}\leq 1\%$$ (5)

$$\delta_{i}(x,y,z)=-MR\cdot (\frac{\sigma_{i}-\bar{\sigma }}{\bar{\sigma }}){\tilde{n}}_{\left(x,y,z\right)}$$ (6)

$$\;MR_{Adaptive}^{\left(k\right)}=MR_{}^{\left(0\right)}\left[\frac{\sigma_{\text{max}}^{\left(k\right)}}{\sigma_{\text{max}}^{\left(0\right)}}\right]$$ (7)

### Design prototype and *in vitro* test

Computerized additive manufacturing has novel potential in prosthetic fabrication [51], but few reports are currently available in dentistry. In this study, the patient jaw model and both the initial and optimized RPDs were prototyped using 3D printing to demonstrate the integration of CAD with CAM. The mandible was printed with a stereolithography machine (Projet HD 3000; 3D Systems, South Carolina, USA) with 16 μm building layer thickness in Shore 90D polyurethane. The mucosa layer was molded onto the rigid bone model, with Shore 15AF polyurethane. As limited by the printable materials commercially available, the metal frames of both dentures were fabricated with stainless steel 316 GP1 by direct metal laser sintering (EOSINT M 270; EOS, Michigan, USA) at 20 μm per building layer. The artificial teeth and denture base were also over-molded with Shore 30D polyurethane.

The prototypes were tested *in vitro* to explore the outcomes of the proposed CAD/CAM procedure with a universal testing machine (Instron 3360; Instron, Victoria, Australia). Two *in vitro* experimental methods were used to examine the contact between the denture base of RPD prototypes and the mandibular residual ridge model. The first was to apply white silicone (Fit Checker II; GC Corporation, New South Wales, Australia) for checking the fit, and the second was using a pressure sensitive film (model 4LW; Fuji Film, New South Wales, Australia) to examining the pressure distribution.

## Results

### Optimization convergence

**Fig 3:**
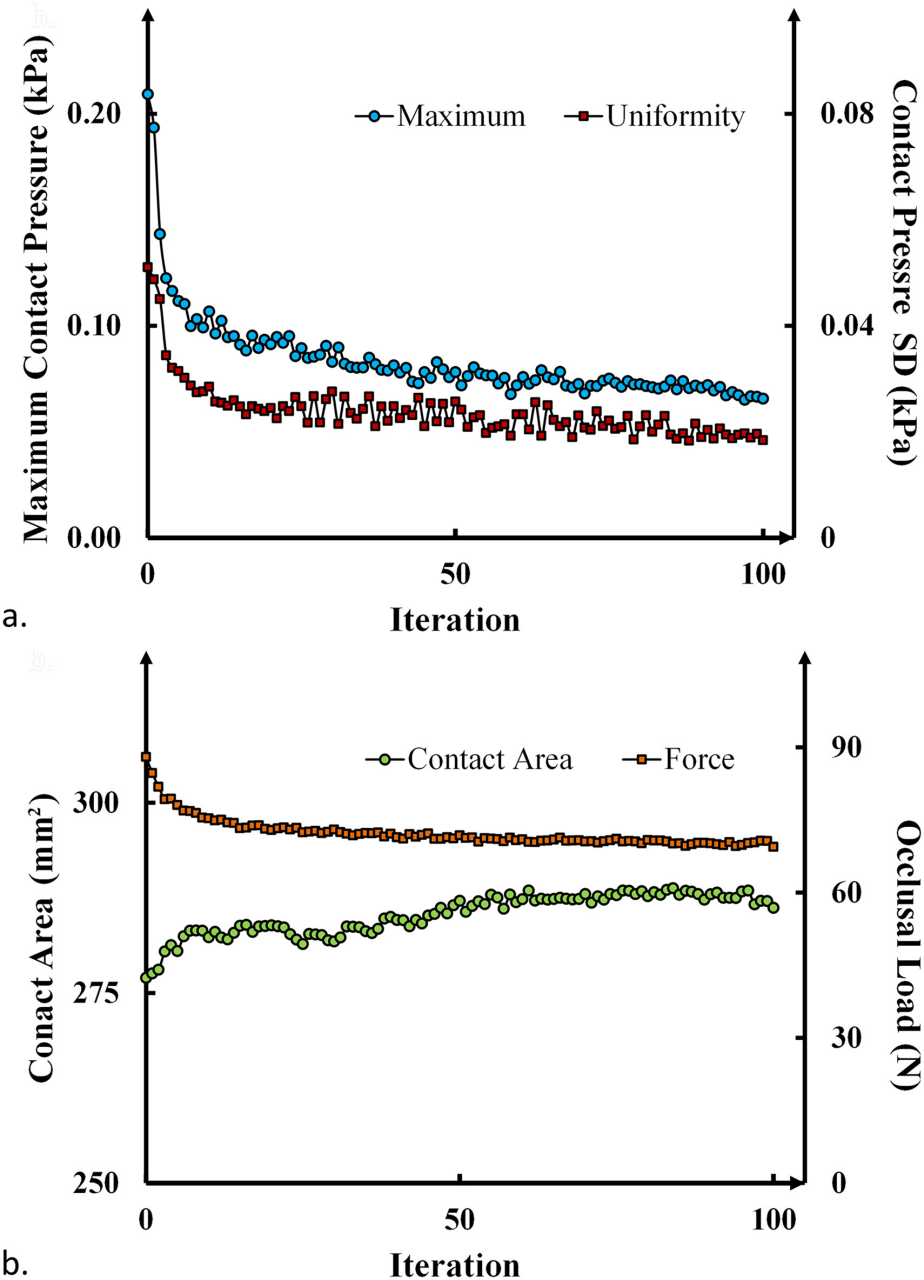
Convergence history, a) the maximum contact pressure and the deviation of contact pressure (i.e. the objective function Eq (5)); b) the contact area and the load transferred from denture base during the optimization.

Both the maximum contact pressure (MAX) and standard deviation (SD) are plotted in Fig 3a against the iteration number of the BESO computational design (Details are included in the S1 Table). Our algorithm and parameters demonstrated a balance between optimization efficiency and robustness of convergence. Through a smooth convergence trend, the optimization reduces the maximum pressure from 209.2 kPa to 65.6 kPa (by 70%), and improves the uniformity from ± 50.3 kPa to ± 18.4 kPa (by 63%). The computational time for 100 iterations of BESO contact optimization is about a day.

The variation of the effective contact area and the total load transferred from the denture base to mucosa tissue are plotted in Fig 3b. An uprising trend shows the increasing effectiveness in the contact surface within a fixed design boundary, and some proportion of the load is shifted from the denture base onto the supportive abutment teeth (premolars) via the clasps and rests.

### Denture modification and contact pressure

**Fig 4:**
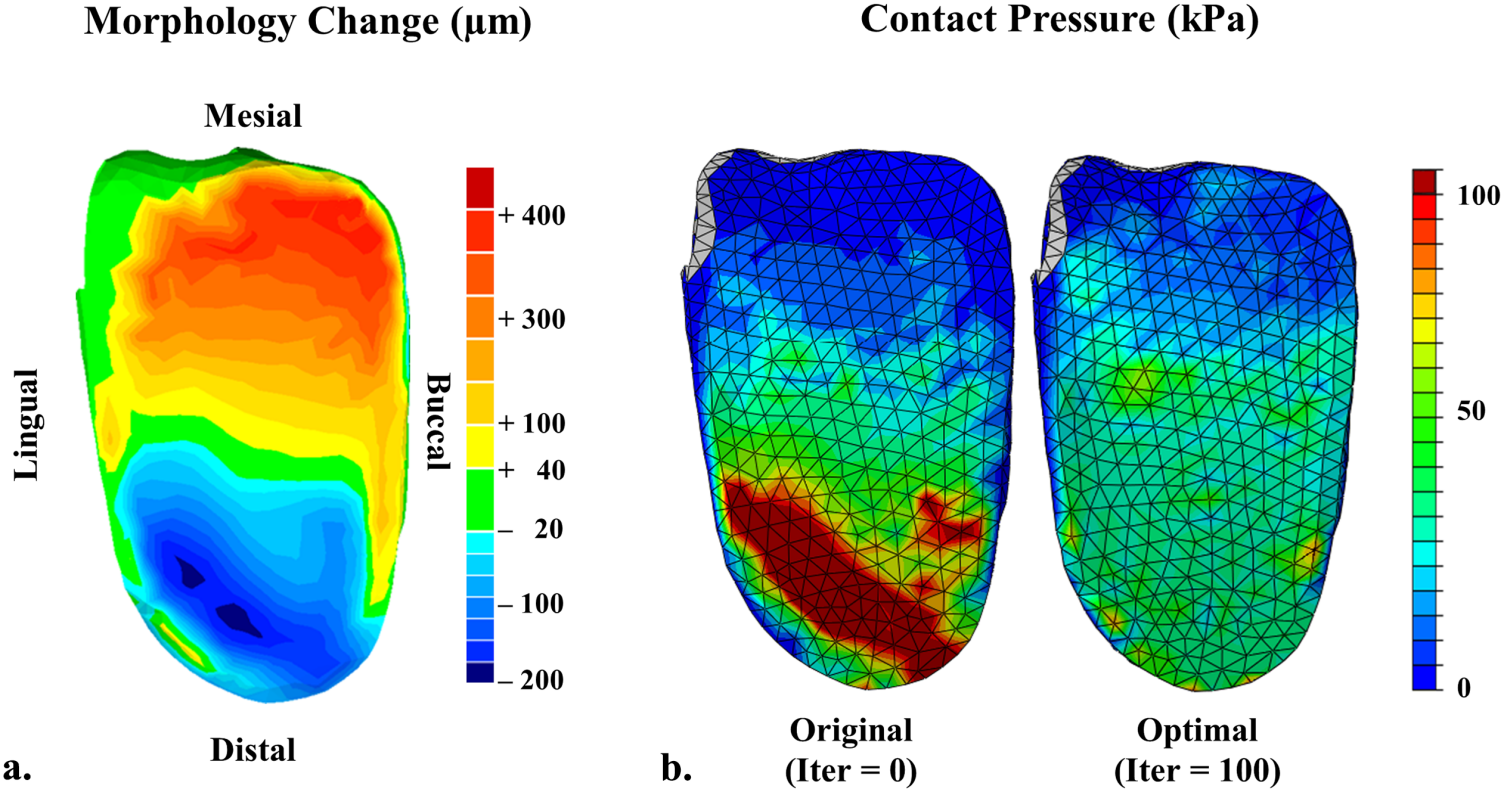
Optimization outcomes, a) the modification made to the denture base through the BESO shape optimization with an adaptive modification rate; the maximum material deposition and removal are 382 and 224 um; b) the contact pressure contours on the initial denture base (left) and the optimized denture base (right).

Fig 4a shows the optimal denture shape with the maximum material addition of 382 μm thick and the maximum removal of 224 μm deep. To reduce the cantilever effect of the initial design, more material is added to the mesial end of the denture base, whereas the thickness is reduced at the distal end. The buccal side of the denture is optimized to share more load in this specific patient case for better efficiency. Fig 4b shows the contours of contact pressures on the denture base before and after the optimization, which clearly exhibit the reduction of pressure concentration severity and improvement in its uniformity. Not only the mesial area, but also the edges of the denture base achieve the improved contact conditions, allowing more effective and smoother load transfer from denture base to mucosa.

### *In vitro* loading test

**Fig 5:**
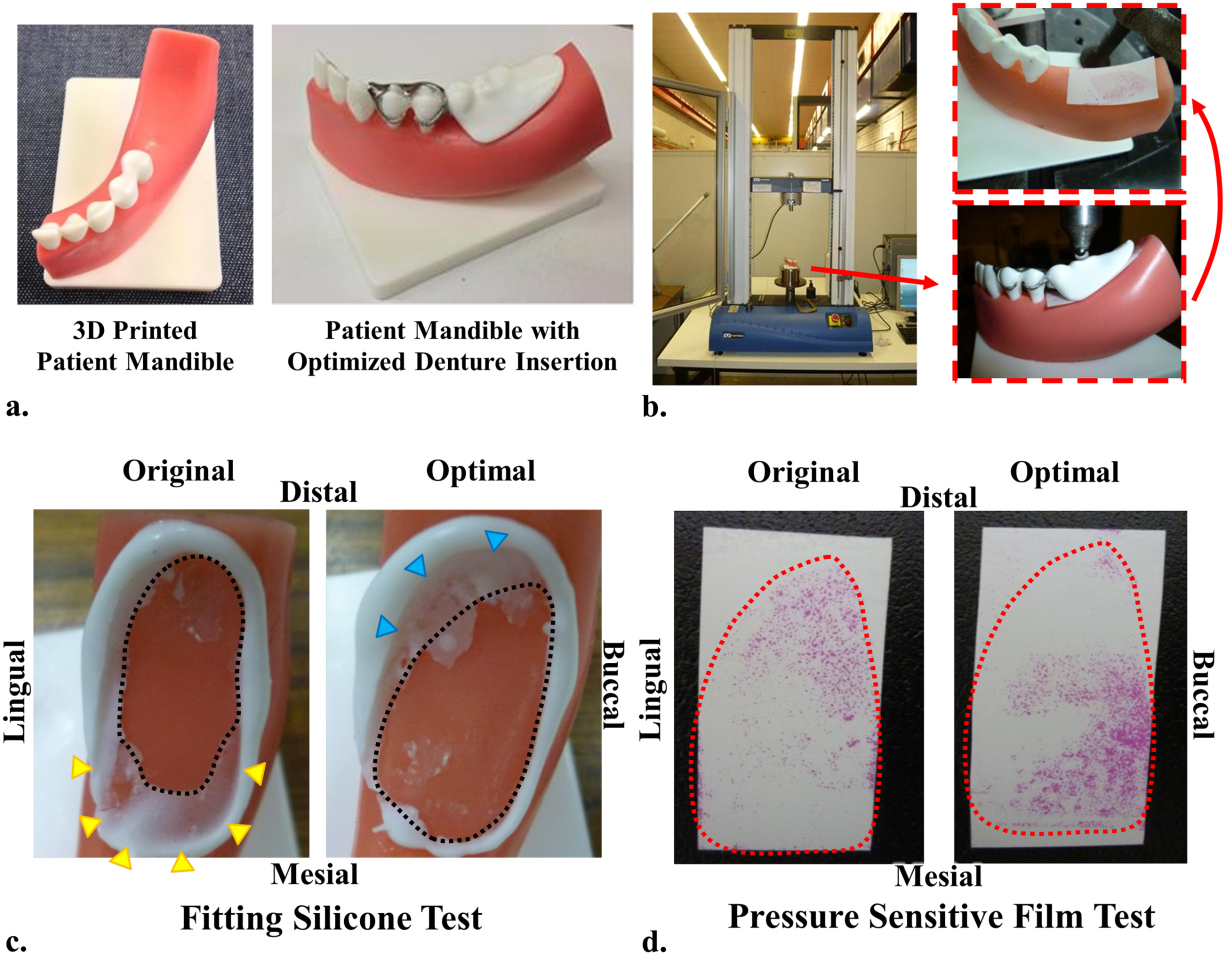
Prototypes and *in vitro* test, a) the subject jaw model (upper) and the optimized denture (lower); b) *in vitro* loading test performed under Instron, with a pressure sensitive firm between the denture and the jaw; c) the fitting white silicone test; d) the pressure sensitive film test.

Fig 5a shows the prototyped mandible (upper) alone and with the optimal denture (lower). Fig 5b illustrates the loading setup for the *in vitro* test, in which the pressure film was inserted between the mandibular residual ridge model and the denture base prototype. The fitting outcomes of white silicone are shown in Fig 5c, and a tight contact in this test squeezed silicone out to create a blank area (black dash line) under loading. Under the initial denture, a medium layer of silicone indicated insufficient load bearing on the mesial end, as highlighted by the yellow triangles. Through the optimization, the supportive contact area was expanded in the mesial direction, while some distal areas became less effective, indicated by the blue triangles, in these models. Consistent trends were observed with the pressure sensitive film test in Fig 5d. The initial denture led to stress concentration at the distal end, and this load was re-distributed towards the mesial direction under the optimized denture. It is noted that although *in vivo* mechanical behavior cannot be quantified precisely through these *in vitro* models, the *in vitro* tests provide meaningful assessment of the optimization outcomes without being influenced by physiological factors.

## Discussion

### Pressure-pain threshold (PPT)

**Fig 6:**
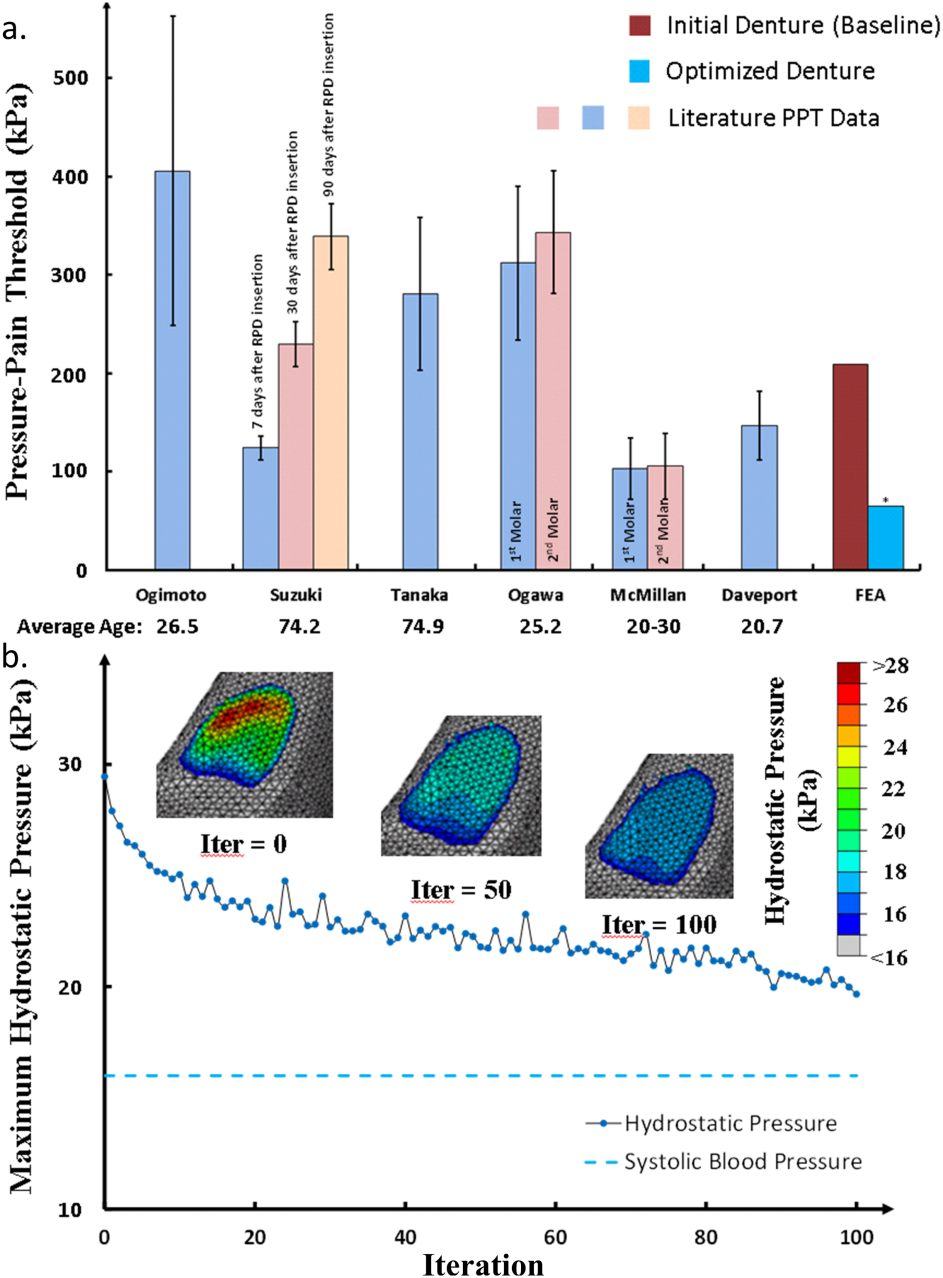
Clinical implications, a) the maximum contact pressures induced by both initial and optimized dentures are compared with the clinical pressure-pain thresholds (PPTs) over the distal region of the mandible. The maximum contact pressure (*) under the optimized denture is significantly lower than the PPTs available from literature (*p* < 0.01). b) Hydrostatic pressure induced by the denture insertion through the optimization procedure, indicating the reduction of disturbance severity to the blood circulation.

The oral mucosa, being a supportive tissue, is found to be mechanically and physiologically responsive to functional pressure during mastication. Previous research has revealed that high contact pressure can trigger pain in the oral mucosa, and a short term concern lies in discomfort and painful sensation for denture users, which is also associated with denture-related stomatitis [52–54]. A pressure-pain threshold (PPT) was defined as the lowest pressure that causes pain [55, 56]; and Fig 6a compares the clinical PPT data with both averages and standard deviations reported in the literature [3, 10, 11, 41, 42, 52, 55–60] along with our FE modeling results (Details are included in the S2 Table). Although the initial FE denture model (baseline) provides a ‘perfect’ morphological match to the mucosa, the maximum contact pressure (209.2 kPa) lies in the mid-range of PPTs. After the optimization, the maximum contact pressure is reduced by nearly 70% (65.6 kPa), which falls well below most PPTs (*p* < 0.01, ANOVA[61], standard deviation _(FE)_ = 0).

### Residual ridge resorption (RRR)

Apart from the immediate pain and discomfort, the long term concern is the consequential residual ridge resorption (RRR), caused by raised interstitial fluid pressure (IFP) or known as hydrostatic pressure inside the mucosa [5, 7, 8]. The aging edentulous mandible is mainly supported by the periosteal plexus of blood vessels; therefore it is very sensitive to a diminished level of circulation under occlusal load, causing reduced nutrient supply to and metabolite removal from the supporting mandibular bone [62]. Experimental observations have shown that 50 kPa pressure can reduce the blood flow rate to only 21% within 5 seconds, and further reduction to 15% after 30 seconds [63]. The continuously loaded epithelial cells and surrounding tissues, undergo an inflammatory response, contributing to a variation in permeability of the mucosal tissue, and further compromising circulation [41, 64]. More recent studies have shown that hydrostatic pressure controls osteogenesis and osteoclastogenesis [65, 66], and RRR has been observed at high hydrostatic pressure regions under certain types of dentures [4].

Fig 6b shows the hydrostatic pressure distribution on the mucosa through the optimization process (Details are included in the S3 Table). The maximum hydrostatic pressure was 29.5 kPa for the initial denture, and it decreased to 19.6 kPa (33.5% reduction) after the optimization, where the green-red color (medium to severe disturbance) disappears. More importantly, the distribution has become more uniform on the residual ridge along both mesial-distal and lingual-buccal directions, indicated by the expanded light blue (low disturbance) regions. The magnitude of the hydrostatic pressure indicates the severity of induced disturbance to local blood circulation, showing that the initial high disturbance along the lingual side is eased and more evenly disturbed over the entire optimized contact surface.

### Clinical implications and limitations

The proposed automatic optimization and fabrication procedure provides an alternative to the conventional manual denture fabrication and fitting, and its advantages can be stated as follows. First, the computerized approaches consider intimate interaction with the oral mucosa and jaw bone, including their anatomical and physiological variations. Early studies have shown that bone is a heterogeneous material with local biomechanical property variations, affecting load transfer and strain [1, 6]. The soft tissue also influences the denture masticatory load distribution in a non-linear manner [3–5]. Second, this automatic procedure demonstrated considerable potential in reducing laboratory cost and dependence on clinical skills and experience for individual patient treatment. Despite the proven effectiveness and clinical predictability, the conventional method is facing severe challenges from the ever-growing aging population and demand for denture treatments with shortage of dentists and dental laboratory staff for its labor-intensive nature [67]. These disadvantages are being gradually expunged, such as the need for a minimum of 4 or 5 treatment visits with additional post-insertion follow-ups, high laboratory expenses and time cost [28]. The proposed computer based optimization and additive fabrication procedure may ameliorate these downsides with customized optimal treatment and reduced cost through a computationally automatic process of scan-design-manufacturing. This approach may also benefit the patients with hypersensitive gag reflexes [68, 69], who are likely to vomit during impression making in the conventional method. Third, computer based approaches allow offering a consistent accuracy and reliable reproducibility with quantitative guidelines, while the conventional clinical denture fabrication, adjustment and correction are often performed manually [70], with heavy reliance on techniques and experience available [16]. Lastly, this optimization algorithm can benefit patients in both short-term and long-term perspectives, as shown in reduction of immediate pain/discomfort and potential residual ridge resorption, as well as fewer recall visits for denture adjustment.

There are a few assumptions and limitations in this study. Following the clinical protocol and radiation dosage available, the applied scanning resolution may affect modeling accuracy. The supporting tooth was considered a rigid body without differentiating its individual hard tissues (dentin and enamel), for their less significant differences comparing to the oral mucosa. The other important soft tissue, periodontal ligament, was not included in this study, for its slight thickness beyond the differentiable CT range, and the associated tooth mobility requires further investigation [71]. It would be interesting to explore the effects of different patients (e.g. loading sites, magnitudes, multiple loads, sulcus depth and width, anatomical abnormalities, etc.) and prosthetic conditions (clasp/rest/guide plane design, denture material), for yielding statistical effectiveness of the proposed methods. It is also significant to take into account the consequential bone apposition and resorption activities, but these are beyond the scope of this study.

As the limitations to our prototypes, there is a certain manufacturing tolerance and materials currently available, leading to differences between the real human oral tissues and the printable model. The in-vitro tests were performed on such prototype models and the results of the contact conditions could only serve as a purpose of demonstrating the integration of CAD/CAM system and effectiveness of the optimization algorithm. Nevertheless, this study showcased a feasible new approach from diagnosed scan, computational modeling and design to additive manufacturing for prosthetic dentistry, thereby providing new technological potential for other dental prosthetic treatments in the future.

## Conclusions

This study proposed a fully automatic design optimization and additive fabrication procedure for RPD. The bi-directional evolutionary structural optimization (BESO) technique removes material from the high contact pressure region to lessen the peak contact stress and adds material to under-loaded regions to enhance loading-bearing capacity. The optimized denture base is able to deliver a more evenly distributed contact pressure and reduce the peak pressure, making them well below PPTs. More importantly, the overloaded region was largely reduced via the optimization and a lower hydrostatic pressure was generated, thereby reducing risk of associated long term bone resorption. This proposed computational optimization and additive fabrication procedure demonstrates considerable promise for other dental prostheses, thereby providing quantitative guidelines and computer aided design and manufacturing (CAD/CAM) in the dental clinic for an individual patient.

## Supporting Information

S1 Table Optimization Convergence History through 100 Iterations. (PDF)

S2 Table Summary of Literature Pressure-Pain Threshold Data and Comparison of FEA Results before and after Optimization. (PDF)

S3 Table Change of Hydrostatic Pressure in the Oral Mucosa through Optimization. (PDF)
