# Supplementary material for: Shape Optimization for Additive Manufacturing of Removable Partial Dentures - A New Paradigm for Prosthetic CAD/CAM
Source: PLoS One. 2015 Jul 10;10(7):e0132552. doi: 10.1371/journal.pone.0132552 (PMC4498620; doi:10.1371/journal.pone.0132552)
Supplement: S1 Table — (PDF) [file pone.0132552.s001.pdf]

S1 Table: Optimisaiton Convergence History through 100 Iterations, including Total Vertical Force (TotalNFz), Contact Area (Carea), Maximum Contact Pressure (CPreMax), Contact Pressure Standard Deviation (CPreStv), Contact Pressure Standard Deviation Change Rate (CPreStv Change Rate), Average Contact Pressure (CPreAve), Maximum Vertical Force (MaxNFz), Average Vertical Force (AverNFz), and Number of Nodes in Contact (#Node in Contact).

Iter	TotalNFz	CArea	CPreMax	CPreStv	CPreStv Change Rate	CPreAve	MaxNFz	AverNFz	#Node in Contact
0	88.00	276.99	0.2092	0.0510		0.0444	0.1551	0.0318	295
1	84.70	277.55	0.1935	0.0487	-4.49%	0.0422	0.1556	0.0305	297
2	81.82	278.05	0.1433	0.0450	-7.27%	0.0405	0.1063	0.0294	297
3	79.28	280.45	0.1224	0.0344	-20.78%	0.0393	0.0986	0.0283	298
4	79.38	281.26	0.1164	0.0321	-4.62%	0.0391	0.0824	0.0282	298
5	78.09	280.51	0.1117	0.0314	-1.24%	0.0383	0.0843	0.0278	298
6	76.90	282.44	0.1102	0.0302	-2.49%	0.0375	0.0815	0.0272	298
7	76.85	283.17	0.0999	0.0287	-2.86%	0.0375	0.0992	0.0271	301
8	76.42	283.20	0.1032	0.0274	-2.47%	0.0370	0.0805	0.0270	301
9	75.48	283.16	0.0992	0.0276	0.34%	0.0365	0.0697	0.0267	301
10	75.34	282.33	0.1067	0.0285	1.76%	0.0365	0.0932	0.0267	301
11	74.87	283.02	0.0964	0.0257	-5.50%	0.0361	0.0911	0.0265	301
12	75.03	282.28	0.1023	0.0255	-0.46%	0.0361	0.0854	0.0266	301
13	74.44	282.03	0.0946	0.0249	-1.12%	0.0359	0.0815	0.0264	301
14	74.35	282.90	0.0951	0.0259	1.96%	0.0356	0.0789	0.0263	301
15	73.33	283.81	0.0911	0.0248	-2.21%	0.0351	0.0689	0.0258	301
16	73.39	283.95	0.0883	0.0233	-2.93%	0.0350	0.0762	0.0258	301
17	73.84	283.02	0.0953	0.0248	3.02%	0.0354	0.0750	0.0261	301
18	73.86	283.72	0.0896	0.0243	-0.98%	0.0351	0.0854	0.0260	301
19	73.16	283.79	0.0934	0.0239	-0.84%	0.0348	0.0973	0.0258	301
20	72.90	283.88	0.0913	0.0245	1.13%	0.0346	0.0789	0.0257	301
21	73.26	283.77	0.0947	0.0226	-3.77%	0.0347	0.0735	0.0258	301
22	73.43	283.59	0.0920	0.0248	4.42%	0.0349	0.0834	0.0259	301
23	72.99	282.72	0.0951	0.0239	-1.82%	0.0346	0.0878	0.0258	301

24	73.34	282.02	0.0857	0.0265	5.21%	0.0347	0.0814	0.0260	302
25	72.45	281.42	0.0894	0.0248	-3.37%	0.0343	0.0710	0.0257	302
26	72.58	282.74	0.0848	0.0217	-6.08%	0.0343	0.0805	0.0257	302
27	72.72	282.68	0.0854	0.0267	9.74%	0.0343	0.0631	0.0257	302
28	72.28	282.59	0.0864	0.0217	-9.72%	0.0342	0.0786	0.0256	302
29	72.51	281.90	0.0905	0.0261	8.60%	0.0341	0.0739	0.0257	302
30	73.02	281.76	0.0830	0.0276	2.83%	0.0345	0.0699	0.0259	302
31	72.48	282.28	0.0897	0.0215	-11.90%	0.0341	0.0732	0.0257	302
32	72.13	283.72	0.0821	0.0266	10.02%	0.0338	0.0638	0.0254	302
33	71.79	283.70	0.0806	0.0236	-5.96%	0.0337	0.0695	0.0253	302
34	72.11	283.62	0.0802	0.0224	-2.19%	0.0338	0.0689	0.0254	302
35	72.30	283.11	0.0803	0.0243	3.61%	0.0338	0.0731	0.0255	302
36	72.22	282.85	0.0850	0.0266	4.63%	0.0338	0.0719	0.0255	302
37	72.39	283.41	0.0819	0.0211	-10.95%	0.0338	0.0692	0.0255	302
38	71.57	284.76	0.0792	0.0248	7.28%	0.0332	0.0729	0.0251	302
39	72.16	285.00	0.0789	0.0221	-5.29%	0.0336	0.0633	0.0253	302
40	71.42	284.57	0.0813	0.0248	5.41%	0.0332	0.0689	0.0251	302
41	71.14	284.57	0.0779	0.0226	-4.44%	0.0330	0.0688	0.0250	302
42	72.03	283.76	0.0802	0.0241	2.95%	0.0334	0.0709	0.0254	302
43	71.50	284.58	0.0736	0.0231	-1.84%	0.0333	0.0631	0.0251	302
44	71.89	284.13	0.0729	0.0264	6.42%	0.0332	0.0533	0.0253	302
45	72.18	285.18	0.0781	0.0210	-10.52%	0.0334	0.0661	0.0253	302
46	71.09	285.40	0.0755	0.0254	8.48%	0.0329	0.0672	0.0249	302
47	71.07	286.19	0.0829	0.0220	-6.63%	0.0327	0.0603	0.0248	302
48	71.41	285.44	0.0794	0.0253	6.44%	0.0329	0.0710	0.0250	302
49	71.19	286.53	0.0757	0.0218	-6.86%	0.0327	0.0671	0.0248	302
50	71.81	287.09	0.0781	0.0257	7.66%	0.0330	0.0704	0.0250	302
51	71.29	285.65	0.0719	0.0241	-3.00%	0.0327	0.0637	0.0250	302
52	71.39	286.43	0.0763	0.0209	-6.34%	0.0327	0.0640	0.0249	302
53	70.49	287.05	0.0805	0.0227	3.52%	0.0324	0.0596	0.0246	302
54	71.22	286.69	0.0775	0.0231	0.76%	0.0327	0.0684	0.0248	302
55	71.15	287.91	0.0766	0.0198	-6.51%	0.0325	0.0573	0.0247	302
56	71.01	287.53	0.0765	0.0207	1.90%	0.0325	0.0746	0.0247	302

57	70.62	286.06	0.0727	0.0210	0.52%	0.0324	0.0582	0.0247	302
58	71.34	287.92	0.0754	0.0214	0.72%	0.0327	0.0649	0.0248	302
59	70.78	286.93	0.0677	0.0193	-4.14%	0.0324	0.0589	0.0247	302
60	71.00	287.33	0.0719	0.0233	7.91%	0.0325	0.0717	0.0247	302
61	70.48	288.47	0.0759	0.0233	-0.03%	0.0322	0.0594	0.0244	302
62	70.41	287.13	0.0727	0.0204	-5.58%	0.0322	0.0700	0.0245	302
63	70.70	287.37	0.0743	0.0255	10.03%	0.0323	0.0578	0.0246	302
64	70.77	287.27	0.0790	0.0192	-12.37%	0.0323	0.0625	0.0246	302
65	70.92	287.35	0.0757	0.0250	11.30%	0.0324	0.0614	0.0247	302
66	71.32	287.49	0.0748	0.0222	-5.42%	0.0326	0.0573	0.0248	302
67	70.71	287.39	0.0782	0.0211	-2.32%	0.0324	0.0725	0.0246	302
68	70.76	287.28	0.0717	0.0218	1.47%	0.0323	0.0611	0.0246	302
69	70.81	287.35	0.0709	0.0190	-5.50%	0.0324	0.0593	0.0246	302
70	70.56	288.01	0.0726	0.0230	7.94%	0.0320	0.0614	0.0245	302
71	70.60	286.87	0.0680	0.0208	-4.48%	0.0324	0.0743	0.0246	302
72	70.32	287.69	0.0717	0.0204	-0.75%	0.0319	0.0656	0.0244	302
73	70.58	287.26	0.0716	0.0238	6.77%	0.0320	0.0622	0.0246	302
74	70.80	287.98	0.0741	0.0211	-5.37%	0.0323	0.0695	0.0246	302
75	71.07	287.85	0.0751	0.0221	1.99%	0.0323	0.0656	0.0247	303
76	70.55	288.44	0.0731	0.0205	-3.07%	0.0320	0.0686	0.0245	303
77	70.64	288.43	0.0712	0.0208	0.60%	0.0322	0.0654	0.0245	303
78	70.55	287.99	0.0738	0.0230	4.14%	0.0319	0.0739	0.0245	303
79	70.16	288.35	0.0723	0.0185	-8.69%	0.0319	0.0600	0.0243	304
80	70.86	287.72	0.0724	0.0210	4.81%	0.0321	0.0757	0.0246	304
81	70.77	288.25	0.0716	0.0231	4.18%	0.0321	0.0651	0.0246	304
82	70.74	287.90	0.0710	0.0200	-6.08%	0.0320	0.0761	0.0246	305
83	70.54	288.55	0.0704	0.0213	2.59%	0.0320	0.0661	0.0244	305
84	70.14	288.76	0.0714	0.0230	3.18%	0.0317	0.0643	0.0243	305
85	70.18	287.91	0.0742	0.0195	-6.83%	0.0318	0.0752	0.0244	305
86	69.66	288.43	0.0701	0.0187	-1.58%	0.0315	0.0543	0.0242	305
87	69.99	288.29	0.0739	0.0197	1.97%	0.0318	0.0678	0.0243	305
88	70.28	287.99	0.0706	0.0183	-2.62%	0.0317	0.0561	0.0244	305
89	70.25	287.26	0.0718	0.0215	6.23%	0.0319	0.0628	0.0245	305

90	70.17	287.96	0.0708	0.0190	-4.94%	0.0317	0.0587	0.0244	305
91	69.96	288.19	0.0721	0.0204	2.68%	0.0317	0.0762	0.0243	305
92	69.77	287.51	0.0695	0.0186	-3.40%	0.0315	0.0703	0.0243	305
93	70.40	287.51	0.0712	0.0194	1.48%	0.0318	0.0545	0.0245	305
94	69.60	287.46	0.0672	0.0189	-0.99%	0.0315	0.0690	0.0242	305
95	69.83	288.34	0.0687	0.0191	0.52%	0.0315	0.0659	0.0242	305
96	70.20	288.45	0.0673	0.0189	-0.57%	0.0317	0.0679	0.0243	305
97	70.37	286.63	0.0651	0.0192	0.64%	0.0319	0.0642	0.0246	305
98	70.71	287.11	0.0667	0.0187	-0.94%	0.0321	0.0780	0.0246	305
99	70.65	287.06	0.0665	0.0188	0.28%	0.0320	0.0597	0.0246	305
100	69.42	286.19	0.0656	0.0185	-0.67%	0.0315	0.0768	0.0243	305
