# Supplementary material for: Shape Optimization for Additive Manufacturing of Removable Partial Dentures - A New Paradigm for Prosthetic CAD/CAM
Source: PLoS One. 2015 Jul 10;10(7):e0132552. doi: 10.1371/journal.pone.0132552 (PMC4498620; doi:10.1371/journal.pone.0132552)
Supplement: S2 Table — (PDF) [file pone.0132552.s002.pdf]

S2 Table: Summary of Literature Pressure-Pain Threshold Data and Comparison of FEA Results before and after Optimization

Name	Age	Number	Load	Load SD	Loading Cell Diameter	Pressure	Pressure SD	Note
Ogimoto	26.5	10	260	100.9	2.0	405.53	157.38	
Suzuki	75	8	358	35.8	6.0	124.08	12.41	7days
	75	8	662	66	6.0	229.45	22.88	30days
	75	8	978	97	6.0	338.98	33.62	90days
Ogawa	27.2	10	380	50	1.5	1053.68	138.64	
Tanaka	74.9	20	180	50	2.0	280.75	77.99	
Ogawa	25.2	20	200	50	2.0	311.94	77.99	1st Molar
	25.2	20	220	40	2.0	343.14	62.39	2nd Molar
McMillan	20-30	10	264	79	4.0	102.94	30.80	1st Molar
	20-30	10	271	86	4.0	105.67	33.53	2nd Molar
Daveport	20.7	10	212	50	3.0	146.96	34.66	
Ori-FEA						209.19		
Optimized-FEA						65.11		
