# Supplementary material for: Shape Optimization for Additive Manufacturing of Removable Partial Dentures - A New Paradigm for Prosthetic CAD/CAM
Source: PLoS One. 2015 Jul 10;10(7):e0132552. doi: 10.1371/journal.pone.0132552 (PMC4498620; doi:10.1371/journal.pone.0132552)
Supplement: S3 Table — (PDF) [file pone.0132552.s003.pdf]

S3 Table: Change of Hydrostatic Pressure in the Oral Mucosa through Optimization

Iter	Hydro (kPa)
0	29.45
1	27.91
2	27.24
3	26.50
4	26.34
5	25.95
6	25.46
7	25.19
8	25.11
9	24.85
10	25.04
11	24.00
12	24.60
13	24.06
14	24.76
15	23.95
16	23.57
17	23.86
18	23.59
19	23.85
20	23.04
21	22.91
22	23.56
23	22.71
24	24.75
25	23.26
26	23.37
27	22.75
28	22.80
29	24.08
30	22.68
31	23.01
32	22.50
33	22.51
34	22.58
35	23.26
36	22.94
37	22.71
38	22.01
39	22.20
40	23.19
41	22.17
42	22.54
43	22.26
44	22.71
45	22.50
46	22.67

47	21.75
48	22.39
49	22.26
50	21.80
51	21.74
52	22.52
53	21.63
54	22.08
55	21.70
56	23.26
57	21.75
58	21.71
59	21.67
60	22.03
61	22.60
62	21.52
63	21.71
64	21.58
65	21.91
66	21.63
67	21.57
68	21.38
69	21.18
70	21.48
71	21.72
72	22.35
73	20.95
74	21.63
75	20.73
76	21.58
77	21.24
78	21.74
79	21.04
80	21.74
81	21.16
82	21.17
83	20.98
84	21.60
85	21.21
86	21.47
87	20.84
88	20.68
89	19.97
90	20.59
91	20.51
92	20.46
93	20.32
94	20.20
95	20.26
96	20.76

97	20.08
98	20.32
99	19.98
100	19.66
